# Dysphagia After Cosmetic Submandibular Gland Botulinum Neurotoxin Type A Injection: A Case Report

**DOI:** 10.3390/healthcare14020235

**Published:** 2026-01-17

**Authors:** Seoyon Yang, You Gyoung Yi

**Affiliations:** Department of Rehabilitation Medicine, Ewha Woman’s University Seoul Hospital, Ewha Woman’s University School of Medicine, 260 Gonghang-daero, Gangseo-gu, Seoul 07804, Republic of Korea; seoyonyang@gmail.com

**Keywords:** incobotulinumtoxinA, submandibular gland injection, cosmetic botulinum neurotoxin type A, dysphagia, silent aspiration, videofluoroscopic swallowing study (VFSS), pharyngeal dysphagia, injection-related complications

## Abstract

**Highlights:**

**What are the main findings?**

- A healthy woman developed severe acute oropharyngeal dysphagia, including silent aspiration, after receiving cosmetic incobotulinumtoxinA injections to both subman-dibular glands.
- Videofluoroscopic swallowing study demonstrated marked pharyngeal-phase impairment at 11 days post-injection, with nearly complete resolution by 5 months following dysphagia rehabilitation.

**What are the implications of the main findings?**

- Even typical cosmetic doses of botulinum toxin to the salivary glands can cause clinically significant swallowing impairment, emphasizing the need for cautious injection technique and consideration of ultrasound guidance to enhance safety.
- Clinicians performing esthetic botulinum toxin procedures should be aware of this potential complication and recognize early symptoms to ensure timely evaluation and management.

**Abstract:**

**Background**: Cosmetic injection of botulinum neurotoxin type A (BoNT/A) into the submandibular glands is increasingly performed to enhance jawline contour. Although generally considered safe, unintended diffusion of the toxin can impair pharyngeal musculature and lead to dysphagia. Severe aspiration-prone dysphagia after esthetic submandibular gland injection has rarely been described. **Case Presentation**: A healthy 37-year-old woman developed acute oropharyngeal dysphagia the day after receiving cosmetic contouring injections with incobotulinumtoxinA (Xeomin^®^), administered to both submandibular glands (20 units per gland, performed without ultrasound guidance). She presented to our rehabilitation medicine clinic 11 days later with severe difficulty swallowing solids and liquids. Her functional oral intake was severely restricted (Functional Oral Intake Scale [FOIS] score 3), and the Eating Assessment Tool-10 (EAT-10) score was 24. Videofluoroscopic swallowing study (VFSS) demonstrated markedly delayed pharyngeal swallow initiation, reduced palatal elevation, poor airway protection, consistent laryngeal penetration, and silent aspiration of thin liquids (Penetration–Aspiration Scale score 8). She underwent diet modification and structured dysphagia rehabilitation. At three months, repeat VFSS showed substantial improvement, with only occasional penetration of large-volume thin liquids, corresponding to FOIS 5 and EAT-10 score 8. By five months, VFSS confirmed complete resolution of penetration and aspiration with normalization of swallowing physiology, reflected by a FOIS score of 7 and EAT-10 score of 1. **Conclusions**: This case demonstrates that cosmetic incobotulinumtoxinA injection into the submandibular glands, particularly when performed without ultrasound guidance, can lead to significant oropharyngeal dysphagia. Clinicians performing esthetic lower-face procedures should be aware of this potential complication and ensure timely swallowing evaluation and rehabilitation when symptoms arise.

## 1. Introduction

Botulinum neurotoxin type A (BoNT/A) injection into the salivary glands is an established treatment for sialorrhea (pathologic drooling), as it temporarily inhibits acetylcholine release at neuroglandular junctions, reducing saliva production [1]. BoNT/A is often injected into the parotid and submandibular glands and can effectively decrease drooling with localized action [2].

More recently, off-label cosmetic use of BoNT/A in the salivary glands has emerged, primarily aimed at esthetic contouring rather than functional symptom control [3,4]. In esthetic practice, relatively low doses are injected to reduce the apparent volume of bulky salivary glands—particularly the submandibular glands—in order to create a smoother and slimmer jawline [3]. Jung et al. specifically described this approach as a means to diminish the visible bulge beneath the mandibular angle and thereby enhance lower facial contour [3].

By contrast, the majority of published safety data regarding salivary-gland botulinum toxin injections originate from its therapeutic use for sialorrhea. In this clinical context, reported adverse effects include xerostomia (dry mouth) and dysphagia (swallowing difficulty) [1,5], which are generally described as rare and mild [6]. More serious complications, such as aspiration pneumonia, have been reported only in a small minority of therapeutic cases [7]. However, comparable safety data for cosmetic salivary-gland injections remain limited, underscoring the need for greater clinical awareness when these procedures are performed for esthetic indications.

Here, we present a case of a patient who developed severe oropharyngeal dysphagia after undergoing BoNT/A injections to the submandibular glands for cosmetic purposes. This case highlights that even procedures considered low risk can lead to serious swallowing impairment, emphasizing the importance of clinician awareness and careful technique.

## 2. Case Presentation

### 2.1. Patient Information

A 37-year-old woman with no significant past medical history received cosmetic BoNT/A injections into both submandibular salivary glands at a local outpatient cosmetic clinic (Table 1). The procedure was performed purely for esthetic purposes to improve lower facial contour, and there was no history of sialorrhea or salivary gland disease. She had no prior neurological disease, head and neck surgery, esophageal disorders, or recent medication changes that could account for her symptoms. She also reported no history of drug or food allergies.

**Figure 1:**
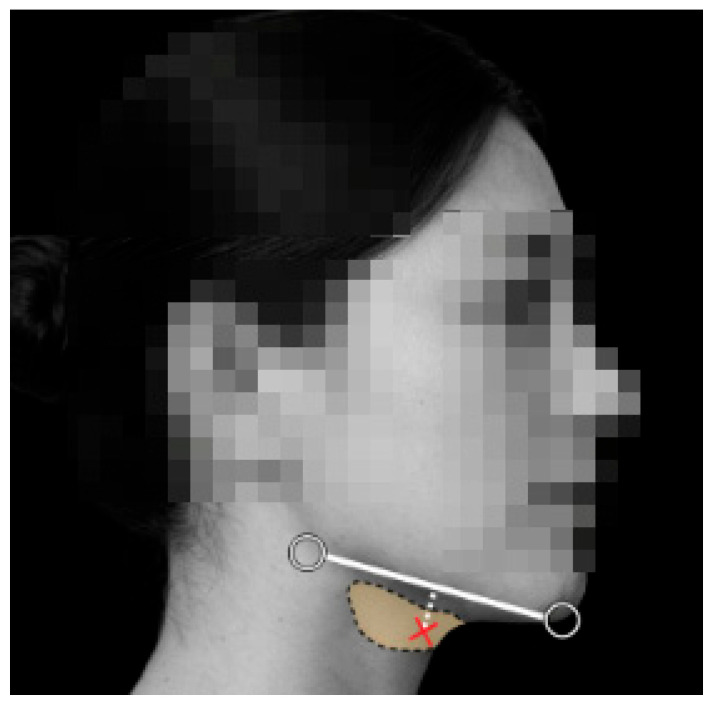
Schematic illustration of the cosmetic BoNT/A injection into the submandibular salivary gland. The shaded area indicates the anatomical location of the submandibular gland beneath the mandible. The injection trajectory (white dotted line) represents an inferior-to-superior approach commonly used in cosmetic practice, with the red cross marking the injection site.

The cosmetic procedure was performed in July 2025, during which incobotulinumtoxinA (Xeomin^®^) was injected into both submandibular glands (Figure 1). A single bolus injection of 20 units was administered into each gland. The injections were performed without ultrasound guidance, relying solely on surface anatomical landmarks. No immediate adverse effects were noted on the day of injection.

### 2.2. Onset of Symptoms

The patient’s swallowing function was normal on the day of injection. However, beginning on post-injection day 1, she noticed an abnormal throat sensation and increasing difficulty swallowing. She described the sensation as “something is stuck and not moving” and initially suspected an upper respiratory infection. Over the following 1–2 days, her symptoms progressed to overt dysphagia affecting both solids and liquids. She experienced frequent choking episodes during meals, difficulty coordinating swallowing, and episodes of nasal regurgitation when drinking water, suggesting impaired palatal function. She denied hoarseness, limb weakness, sensory changes, or cognitive symptoms. By post-injection day 11, her oral intake had become markedly restricted, prompting her to present to our outpatient rehabilitation medicine clinic for evaluation. At presentation, her functional oral intake corresponded to a Functional Oral Intake Scale (FOIS) score of 3 [8], and she had lost approximately 3 kg due to reduced oral intake. Her Eating Assessment Tool-10 (EAT-10) score was 24 [9], indicating severe subjective swallowing impairment.

### 2.3. Diagnostic Assessment

Neurologic examination demonstrated normal cranial nerve function, with the exception of delayed swallow initiation. Tongue mobility, lip closure, gag reflex, and speech articulation were intact. Volitional swallowing was impaired, with frequent coughing episodes and difficulty clearing thin liquids. Mild bilateral swelling was palpable over the submandibular gland regions.

**Figure 2:**
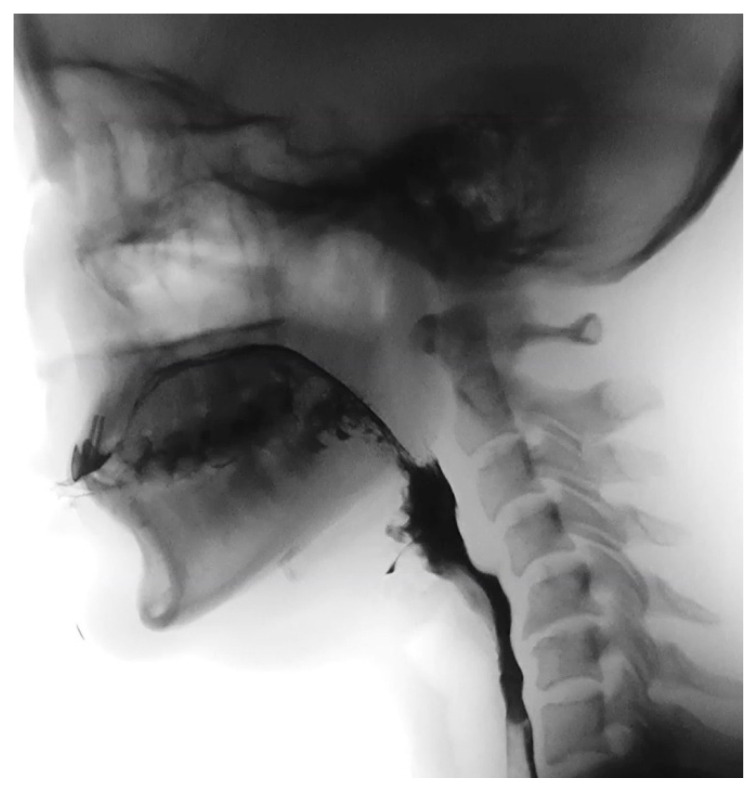
Videofluoroscopic swallowing study (VFSS) performed on post-injection day 11 demonstrating severe oropharyngeal dysphagia. With a semisolid bolus (4 mL), contrast material is retained in the valleculae and pyriform sinuses with impaired laryngeal elevation and epiglottic inversion, and the bolus enters the laryngeal vestibule, consistent with penetration (Penetration–Aspiration Scale [PAS] score 5).

A videofluoroscopic swallowing study (VFSS) was performed on post-injection day 11 to evaluate swallowing physiology (Figure 2). VFSS revealed severe oropharyngeal dysphagia predominantly affecting the pharyngeal phase. Oral preparation and bolus propulsion were preserved, with no premature bolus loss or significant oral residue. In contrast, swallow initiation was markedly delayed across all tested consistencies. Soft palate elevation was reduced, although no frank nasopharyngeal reflux was observed. Laryngeal elevation and epiglottic inversion were impaired, resulting in compromised airway protection. Moderate residue accumulated in both the valleculae and pyriform sinuses following swallowing.

During VFSS, semisolid boluses (4 mL) repeatedly entered the laryngeal vestibule without passing below the vocal folds, corresponding to a Penetration–Aspiration Scale (PAS) score of 5, whereas thin liquid barium (4 mL) resulted in silent aspiration, corresponding to a PAS score of 8 [10]. Within the fluoroscopic field, no structural abnormalities or significant esophageal transit delay were identified. Compensatory strategies, including the chin-tuck maneuver and effortful swallow, did not improve swallowing safety. Due to repeated aspiration events, the VFSS was terminated early for patient safety. Fiberoptic endoscopic evaluation of swallowing (FEES) was not performed because the patient declined the procedure after explanation, and VFSS was considered sufficient for evaluating aspiration risk and guiding management in this case.

### 2.4. Management

The patient was managed with a comprehensive, staged dysphagia rehabilitation program in an outpatient setting, supervised by a qualified speech–language pathologist and rehabilitation physician. The treatment approach was based on commonly used clinical dysphagia rehabilitation protocols reported in the literature [11,12] and adapted to the patient’s tolerance and functional status. Dietary modification was initiated immediately to minimize aspiration risk. The patient was advised to maintain a soft or semisolid diet (e.g., porridge or pureed foods) and to avoid thin liquids unless modified. Small-volume water intake was permitted with strict volume control using a teaspoon or straw, as larger boluses consistently resulted in aspiration during VFSS. Compensatory postural strategies, including the chin-tuck maneuver, were instructed, although these did not significantly improve swallowing safety during instrumental assessment.

Swallowing rehabilitation was conducted three times per week on an outpatient basis and progressed in a staged manner [11] according to clinical improvement. In the early phase, therapy emphasized compensatory techniques and safe swallowing strategies, including effortful swallowing and double swallowing to facilitate clearance of pharyngeal residue. Oromotor facilitation exercises targeting the lips, tongue, and oral cavity were also performed.

As airway safety improved and overt aspiration events decreased, therapy transitioned to a strengthening-focused phase. Exercises targeting hyolaryngeal elevation and pharyngeal contraction were introduced. The Shaker exercise (head-lift exercise) [13] was prescribed to strengthen the suprahyoid musculature, performed under supervision and continued as a home exercise program. The Masako maneuver (tongue-hold swallow) [14] was practiced to enhance posterior pharyngeal wall contraction, performed without bolus intake for safety. Effortful swallows were continued throughout the rehabilitation period to reinforce pharyngeal pressure generation.

Each therapy session lasted approximately 30–40 min, with exercises performed in sets consistent with commonly reported clinical protocols. Patients were encouraged to perform selected exercises daily at home as tolerated, with adherence and proper technique reinforced during outpatient visits. In addition, surface neuromuscular electrical stimulation (sNMES) [15] was applied as an adjunctive therapy during selected sessions. Stimulation was delivered using a VitalStim^®^ device (Chattanooga Group, Hixson, TN, USA), employing a biphasic pulsed current at a frequency of approximately 80 Hz and a pulse duration of 300 μs. Stimulation intensity was gradually increased to achieve visible and tolerable muscle contraction. Electrodes were placed in the submental region targeting the suprahyoid musculature, and stimulation was applied for approximately 20–30 min per session, in conjunction with active swallowing exercises, consistent with established clinical practice [16,17].

Supportive care included education on oral hygiene and adequate hydration to manage thickened secretions related to reduced salivary flow. No pharmacologic treatment for dysphagia was administered during this period, and the patient did not receive anticholinergic or pro-motility agents. Over the subsequent weeks, the patient demonstrated gradual clinical improvement, with a reduction in choking episodes and progressive advancement of oral intake. By August 2025, she was able to tolerate most solid foods, although thin liquids continued to require caution. The patient did not develop aspiration pneumonia or other dysphagia-related medical complications during the follow-up period. She reported mild anxiety related to swallowing difficulties in the acute phase; however, symptoms did not require pharmacologic treatment, and she declined referral to psychiatric services. These symptoms gradually resolved as swallowing function improved.

### 2.5. Follow-Up VFSS at 3 Months

At approximately 12 weeks after the BoNT/A injections, a repeat VFSS was performed to objectively assess her progress. This study showed marked improvement in swallowing function compared to the first VFSS (Table 2). The pharyngeal transit time had normalized—there was no longer a significant delay in triggering the swallow reflex for any consistency. The soft palate elevation and laryngeal elevation during swallows were better, resulting in effective airway protection. No aspiration was observed during the study. There was no penetration for semisolid and solid boluses; even with thin liquids, nearly all trials were swallowed safely. Only on one occasion, with an 8 cc rapid spoonful of liquid barium, a trace penetration was observed, in which a small amount of contrast briefly entered the laryngeal vestibule but was immediately ejected without descending below the vocal folds (PAS 2). [10]. The patient was able to clear any mild residue with a spontaneous second swallow, and essentially no significant residue remained in the valleculae or pyriforms. These findings indicated a near-complete recovery of her swallowing mechanism. She reported that she had resumed a normal diet by this time, though she still exercised caution with very thin liquids (preferring water in small sips). Her functional oral intake improved to FOIS 5, and her EAT-10 score decreased to 8, reflecting moderate residual symptoms but significant clinical improvement.

### 2.6. Outcome at 5 Months

**Figure 3:**
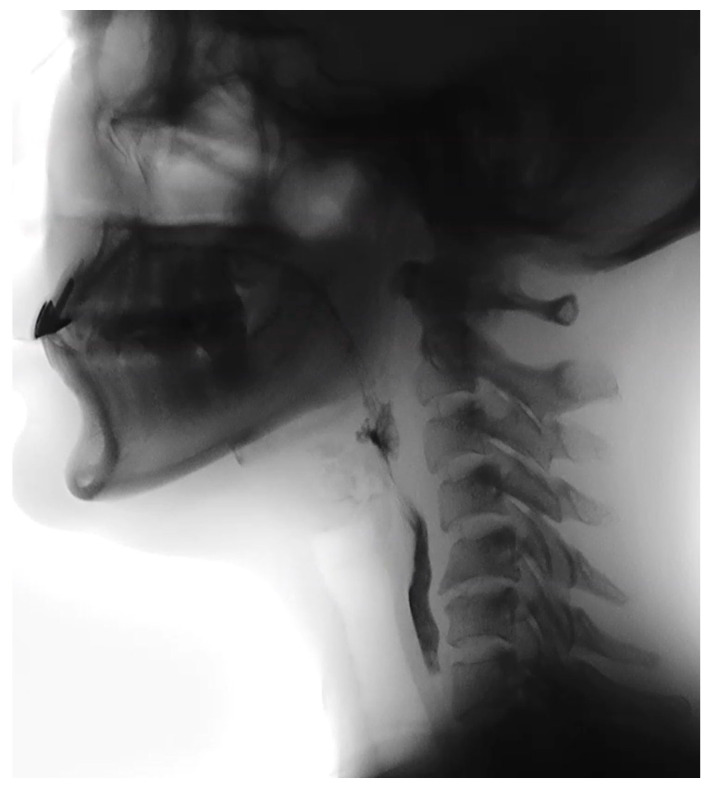
Follow-up videofluoroscopic swallowing study with 8 mL thin liquid barium, demonstrating intact airway protection without penetration or aspiration.

By 5 months after the BoNT/A injections, the patient reported her swallowing was “back to normal.” A third VFSS at 5 months post-injection confirmed near-complete recovery (Table 2). There was no penetration or aspiration observed even with consecutive swallows of unthickened liquid (Figure 3), and all food consistencies were swallowed safely. Residue was absent, and all compensatory strategies were no longer needed. She fully returned to a regular diet and unrestricted fluid intake, corresponding to FOIS 7, and her EAT-10 score was 1, indicating minimal subjective difficulty. Nighttime coughing and throat clearing had resolved, and she had resumed normal occupational and social activities.

## 3. Discussion

This case highlights the potential for BoNT/A injected in the head and neck region to cause unintended muscle weakness beyond the target tissue. Although FEES can provide detailed information on pharyngeal secretion management and laryngeal sensation, it was not performed in this case because the patient declined the procedure. VFSS was selected as the primary assessment modality, as it allowed comprehensive evaluation of bolus transit, airway invasion, and aspiration severity across different consistencies, which was sufficient for clinical decision-making in this patient. In our patient, the intended targets were the submandibular glands, but the resulting dysphagia suggests that nearby muscles critical for swallowing were affected. There are a few plausible mechanisms: (1) Local diffusion of toxin: BoNT/A can spread from the injection site into adjacent tissues if not precisely placed. The floor of mouth and upper pharyngeal constrictor muscles lie near the salivary glands; accidental diffusion or intramuscular injection could temporarily paralyze these muscles [18]. Weakness in the suprahyoid muscles (which elevate the larynx) and pharyngeal constrictors would explain the reduced laryngeal elevation and slow clearance seen on VFSS. (2) Excessive saliva reduction: Even if the toxin stays within the glands, the profound reduction in saliva volume can itself impair swallowing. Saliva is critical for lubrication and initiating the swallow reflex; an extremely dry mouth (xerostomia) leads to difficulty in forming a cohesive bolus and can blunt the reflexive swallow trigger [18]. Thick, viscous mucus replacing normal saliva was noted in similar cases [7] and can contribute to the sensation of something “stuck” and to penetration events. In reality, both factors likely contributed in this case—the patient had both signs of muscular incoordination and symptoms of dryness.

BoNT/A injection into salivary glands for medical reasons (drooling) has been studied more extensively than for cosmetics. Overall, the literature suggests that dysphagia is an uncommon but recognized complication of this treatment. In a case report by Layton et al., a 26-year-old cerebral palsy patient developed significant oropharyngeal dysphagia after three rounds of intraglandular BoNT/A for sialorrhea [7]. The patient experienced thick secretions, choking, and required treatment for aspiration pneumonia, leading the clinicians to discontinue BoNT/A despite its efficacy in drying up drooling. Larger series have quantified the risk in pediatric populations with neurologic disorders: Van Eck et al. reported transient oral-motor side effects in about 33–36% of children after four-gland (parotid + submandibular) BoNT/A injections for drooling [19]. These effects included difficulties with swallowing, chewing, or speaking, but most were mild and resolved within a month [19].

On the other hand, more severe outcomes, while rare, have been documented. A long-term pediatric cohort study of salivary gland BoNT/A injections found major complications in approximately 4% of cases, including instances of severe dysphagia and aspiration pneumonia requiring hospitalization [20]. Notably, those injections were given to children, many of whom already had baseline dysphagia or risk of aspiration due to their neurological condition. Our patient differs in that she was a young, healthy adult with no pre-existing dysphagia—yet she experienced a level of swallowing impairment similar to those severe cases. To our knowledge, reports of such complications in the cosmetic use of salivary gland BoNT/A are scarce. This may be due to underreporting or the fact that cosmetic doses are typically smaller; however, it highlights that even in healthy individuals, significant dysphagia can occur if the toxin affects the swallowing muscles.

Proper technique in administering glandular BoNT/A is crucial to minimize unwanted effects. Although no universally accepted guideline exists specifically for cosmetic BoNT/A injection into the submandibular glands, several published cosmetic protocols provide practical recommendations regarding dose, injection points, and guidance methods (Table 3). Reported cosmetic dosing typically ranges from 15 to 20 units per submandibular gland, administered either as a single intraglandular depot or divided among multiple injection points, with most authors emphasizing the importance of ultrasound guidance to ensure accurate intraglandular placement and to minimize unintended diffusion to adjacent swallowing-related musculature [18]. In our case, the injections were performed “blind,” which might have increased the chance of misplacement. Additionally, dividing the dose among multiple small injection points within the gland (rather than a single large bolus) can help contain the spread. The total dose used for cosmetic gland reduction is not standardized—it may be similar to doses used for drooling (for example, a typical regimen for sialorrhea in adults is 100 units of BoNT/A split as 30 units to each parotid and 20 units to each submandibular [18]. If an excessive dose was injected or if the patient had an increased sensitivity, systemic spread could occur. Patient factors should also be considered: any underlying subclinical dysphagia or anatomical variant could make a person more susceptible to the effects of saliva reduction. In this patient, there were no such factors identified, suggesting the complication was purely injection-related. Practitioners performing cosmetic “salivary gland slimming” should carefully weigh the benefits against these potential risks. It would be prudent to inform patients pre-procedure that although rare, they might experience dry mouth or difficulty swallowing for some weeks after the injection [18]. In patients who use their voice professionally or have any swallowing complaints, extra caution is warranted.

Given that no specific antidote currently exists for BoNT/A once it has bound at the neuromuscular junction, management of BoNT/A-induced complications remains largely supportive until neuromuscular function recovers. This limitation represents a clinically important unmet need, particularly in cases of significant adverse effects such as dysphagia or respiratory compromise. Ongoing research has explored potential pharmacological countermeasures aimed at inhibiting botulinum neurotoxin activity after exposure. Experimental approaches include the development of small-molecule inhibitors targeting the light chain of BoNT/A, which functions as a zinc-dependent endopeptidase responsible for cleaving SNAP-25 and blocking acetylcholine release. Computational and in silico screening studies have identified candidate compounds with predicted inhibitory activity against the BoNT/A light chain; however, these agents remain at a preclinical stage and are not yet available for clinical use [23]. Until such antidotes or post-exposure therapies become clinically viable, early recognition of toxin-related complications and timely initiation of supportive measures remain the cornerstone of management.

Therefore, management is focused on supportive care and preventing complications. Key aspects include dietary modifications (as implemented in this case) to prevent aspiration. The use of thickeners for liquids and a texture-modified diet can dramatically reduce aspiration events in oropharyngeal dysphagia. Swallow therapy exercises may help accelerate functional recovery by promoting neural plasticity and strengthening compensatory muscle activity, though evidence for exercise specifically counteracting BoNT/A effects is anecdotal. In our patient, a combination of exercises (Shaker, effortful swallow, etc.) was used alongside cautious oral intake. These dysphagia exercises are targeted movements that improve the strength, coordination, and timing of the muscles used in swallowing, aiming for long-term functional improvement [24,25]. For instance, exercises that target the suprahyoid musculature (such as the Shaker exercise or chin tuck against resistance) [25,26] can increase hyolaryngeal elevation and have been shown to reduce aspiration in patients with swallowing impairments. By regularly performing such exercises, our patient likely facilitated neuromuscular re-education and compensatory strengthening while waiting for neural recovery, potentially shortening the duration of severe dysphagia [27]. We observed that her swallowing function gradually improved over the course of 3–5 months, corresponding with the expected timeline of nerve terminal sprouting and return of acetylcholine release as the botulinum toxin effect faded [28]. Notably, this recovery timeline (~3–5 months) is at the favorable end of the typical recovery range for BoNT/A-induced deficits (which can span weeks to months). It is conceivable that the prompt initiation of rehabilitative therapy contributed to this favorable outcome by expediting functional improvements in swallowing. If her dysphagia had been any worse (e.g., frank aspiration of even thick liquids or an inability to swallow saliva), we would have considered placing a nasogastric feeding tube for temporary nutritional support until improvement occurred. Close monitoring is essential: we performed serial VFSS to guide our recommendations, and this objective tracking helped determine when it was safe for her to liberalize her diet.

The patient’s positive outcome with full recovery reinforces some important clinical principles. First, early recognition and intervention in BoNT/A-related dysphagia are paramount. Even in an otherwise healthy individual, significant dysphagia can cause serious complications like aspiration pneumonia if not managed properly, as documented in other cases [20]. Thus, clinicians should maintain a high index of suspicion for swallowing difficulties after head and neck BoNT/A injections and promptly refer such patients for a swallowing evaluation (e.g., by speech–language pathology) if symptoms arise. Second, aggressive supportive care and rehabilitation should be initiated as soon as dysphagia is identified. In similar future cases, we advise implementing diet modifications and a tailored swallowing exercise program to maintain nutrition and airway safety while the toxin’s effects subside. This case suggests that proactive rehabilitation may help patients recover more quickly and avoid complications, serving as a guide for managing BoNT/A-induced dysphagia. Finally, thorough patient counseling and meticulous injection technique (ideally with ultrasound guidance) are essential preventive strategies. Patients undergoing off-label cosmetic BoNT/A injections in the salivary glands should be informed about potential side effects like dry mouth or transient dysphagia so that they can seek care early if these occur. In summary, this case demonstrates that with vigilant supportive management—including diet modifications, close monitoring, and rehabilitative swallowing therapy—patients can safely recover fully as the neurotoxin effects resolve, even after a significant adverse event.

## 4. Conclusions

Cosmetic injection of BoNT/A into the submandibular salivary glands is an emerging procedure for facial slimming, but it is associated with potential procedure-related risks. This case illustrates that even in a young and healthy individual, such injections can lead to significant oropharyngeal dysphagia, likely through a combination of toxin diffusion to swallowing muscles and extreme reduction in saliva. The resultant dysphagia can cause aspiration and adversely affect nutrition and quality of life, albeit temporarily. With vigilant supportive management—particularly timely swallowing rehabilitation along with diet modification and follow-up swallow studies—patients can recover fully as the neurotoxin effects resolve. Clinicians are advised to use a precise technique (preferably ultrasound guidance) to minimize inadvertent spread, and to counsel patients on potential side effects like dry mouth or swallowing difficulty before undertaking this off-label cosmetic treatment. Early recognition and intervention in cases of post-BoNT/A dysphagia are paramount to prevent complications. This case adds to the body of evidence reminding practitioners that no procedure is truly minor—even common cosmetic injections can have uncommon but serious sequelae that should be acknowledged and managed with care.

## Figures and Tables

**Table 1:** Patient and Procedure Details.

**Item**	**Details**
Patient	37-year-old woman
Relevant medical history	No prior neurologic disease, neck surgery/radiation, esophageal disorder, or baseline dysphagia
Drug allergy history	None reported
Indication	Cosmetic lower-face contouring (submandibular gland volume reduction)
Setting	Local cosmetic clinic (outpatient)
Toxin formulation	IncobotulinumtoxinA (Xeomin^®^)
Guidance method	No ultrasound guidance (landmark-based)
Total dose	40 units (bilateral glands)
Dose per gland	20 units per submandibular gland
Dose per injection site	20 units/site (single-depot injection, one injection point per gland)
Dilution/injection volume	Not available (patient could not confirm)
Immediate post-procedure issues	None on the day of injection

**Table 2:** Longitudinal changes in swallowing function and VFSS findings.

**Time Point**	**FOIS**	**EAT-10**	**VFSS Key Findings**
Post-injection Day 11 (Initial evaluation)	3	24	Severe oropharyngeal dysphagia; markedly delayed swallow initiation; reduced palatal elevation; impaired laryngeal elevation and epiglottic inversion; consistent laryngeal penetration with semisolid bolus (PAS 5); silent aspiration with thin liquid (PAS 8); moderate vallecular and pyriform sinus residue
3 Months Post-injection	5	8	Normalized swallow initiation; improved palatal and laryngeal elevation; no aspiration; trace penetration with thin liquid on one trial only (PAS 2); minimal residue, cleared with spontaneous second swallow
5 Months Post-injection	7	1	Normal swallowing physiology; no penetration or aspiration across all consistencies; no residue

Abbreviations: FOIS, Functional Oral Intake Scale; EAT-10, Eating Assessment Tool-10; PAS, Penetration–Aspiration Scale; VFSS, videofluoroscopic swallowing study.

**Table 3:** Published Cosmetic Protocols/Recommendations for Submandibular Gland BoNT/A Injection and Comparison with Current Case.

**Author (Year)**	**Toxin Formulation(s)**	**Suggested Dose (Per Gland)**	**Injection Points/Dose per Point**	**Guidance/Injection Site Notes**	**Comparison with Current Case**
Jung et al., 2019 [3]	BoNT/A (onabotulinumtoxinA)	20 units/gland	5 points/gland (≈4 units per point), high concentration reported	Ultrasound-guided intraglandular placement described	Same per-gland dose, but our case used single-point injection and no ultrasound
Karapantzou et al., 2020 [21]	BoNT/A or Xeomin	15 units/gland	Single injection technique (one depot/gland)	Ultrasound guidance emphasized for accurate intraglandular injection	Our dose (20 units/gland) is higher than this protocol; our technique matched “single depot” but without ultrasound
Gelezhe et al., 2023 [22]	(Technique paper; not dose-specific)			Landmark-only placement varies; authors emphasize real-time ultrasound to optimize depth and avoid off-target placement	Supports that lack of ultrasound in our case may increase off-target risk

Abbreviations: BoNT/A, botulinum neurotoxin type A; Xeomin^®^, incobotulinumtoxinA. Note: To date, no formal international guideline exclusively for cosmetic submandibular gland BoNT/A injection was identified; therefore, this table summarizes peer-reviewed cosmetic protocols/technique reports and safety-focused anatomical recommendations.

## Data Availability

The data presented in this study are not publicly available due to privacy and ethical restrictions. Clinical imaging and videofluoroscopic swallowing study data contain identifiable patient information and cannot be shared. De-identified data may be made available from the corresponding author upon reasonable request and with institutional approval.

## Abbreviations

The following abbreviations are used in this manuscript:

BoNT/A	Botulinum neurotoxin type A
VFSS	Videofluoroscopic swallowing study
PAS	Penetration–Aspiration Scale
FOIS	Functional Oral Intake Scale
EAT-10	Eating Assessment Tool-10
sNMES	surface neuromuscular electrical stimulation
